# Revisional One-Step Bariatric Surgical Techniques After Unsuccessful Laparoscopic Gastric Band: A Retrospective Cohort Study with 2-Year Follow-up

**DOI:** 10.1007/s11695-023-07039-7

**Published:** 2024-01-17

**Authors:** Mohamed Hany, Ahmed Zidan, Mohamed Ibrahim, Ahmed Sabry, Ann Samy Shafiq Agayby, Mohamed Mourad, Bart Torensma

**Affiliations:** 1 Department of Surgery, Medical Research Institute, Alexandria University, 165 Horreya Avenue, Hadara, Alexandria, 21561 Egypt; 2 Consultant of Bariatric Surgery at Madina Women’s Hospital, Alexandria, Egypt; 3 Department of Surgery, Faculty of Medicine, Alexandria University, Alexandria, Egypt; 4 Clinical Epidemiologist, Leiden University Medical Center (LUMC), Leiden, the Netherlands

**Keywords:** Revisional surgery, Roux-en-Y gastric bypass, Laparoscopic one anastomosis gastric bypass, Laparoscopic sleeve gastrectomy, Laparoscopic adjustable gastric band, Food tolerance, Percentage of excess weight loss

## Abstract

**Background:**

Laparoscopic adjustable gastric banding (LAGB) has high reported rates of revision due to poor weight loss (WL) and high complication rates. Yet, there is yet to be a consensus on the best revisional procedure after unsuccessful LAGB, and studies comparing different revisional procedures after LAGB are still needed.

**Methods:**

This was a retrospective cohort study that compared the outcomes of one-step revisional Roux-en-Y gastric bypass (rRYGB), one-anastomosis gastric bypass (rOAGB), or laparoscopic sleeve gastrectomy (rLSG) after LAGB. WL, complications, resolution of associated medical conditions, and food tolerance were assessed with a post hoc pairwise comparison one-way analysis of variance (ANOVA) throughout a 2-year follow-up.

**Results:**

The final analysis included 102 (rRYGB), 80 (rOAGB), and 70 (rLSG) patients. After 2 years, an equal percentage of excess weight loss was observed in rOAGB and rRYGB (both >90%; *p*=0.998), significantly higher than that in rLSG (83.6%; *p*<0.001). In our study, no leaks were observed. rRYGB had higher complication rates according to the Clavien-Dindo classification (10.8% vs. 3.75% and 5.7% in rOAGB and rLSG, respectively, *p*=0.754), and re-operations were not statistically significant. Food tolerance was comparable between rOAGB and rRYGB (*p* = 0.987), and both had significantly better food tolerance than rLSG (*p*<0.001). The study cohorts had comparable resolution rates for associated medical problems (*p*>0.60).

**Conclusion:**

rOAGB and rRYGB had better outcomes after LAGB than rLSG regarding WL, feasibility, food tolerance, and safety. rOAGB had significantly higher rates of nutritional deficiencies.

**Graphical Abstract:**

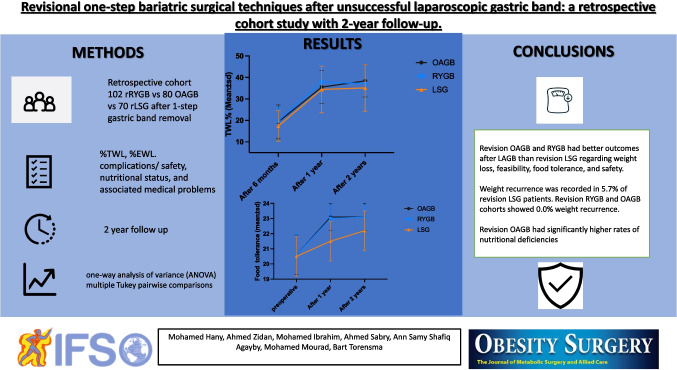

## Introduction

Laparoscopic adjustable gastric banding (LAGB) gained considerable popularity in the early 2000s to become the second most performed procedure in the USA after Roux-en-Y gastric bypass (RYGB) in 2011 with 55,932 procedures. However, it lost popularity throughout the following years, recording 2393 procedures in 2020 [1, 2].

Poor weight loss and high complication rates have been reported in the long term after LAGB, with a reported mean percentage of excess weight loss (%EWL) of 49.1%±13.1% after 10 years and long-term complications and reoperation rates reaching up to 52.9% and 66.1%, respectively [3]. High rates of revisional surgery after LAGB have been reported. Band removal was reported in around 22.9% (5.4–54.0%) of patients, mainly due to band-related complications, and band removal alone formed 27.6% of all revisions in the USA in 2018 [3, 4]

Unique complications for the LAGB have been reported in the literature, with collectively reported rates reaching up to 19%, such as gastric perforations/erosions, migration, slippage, gastric necrosis, esophageal dilatation, and port problems [5, 6]. A revisional procedure should be offered to patients with unsuccessful weight loss (WL), and even patients who need band removal for complications without unsuccessful WL would need a revisional procedure later for the high incidence of weight recurrence (WR) and relapse of associated medical problems [7]. The most reported revisional procedures after LAGB are Roux-en-Y gastric bypass (RYGB) and laparoscopic sleeve gastrectomy (LSG) [8–10]. Recently, some studies have suggested the efficacy and safety of laparoscopic one anastomosis gastric bypass (OAGB) as a revisional option [11]. RYGB after LAGB (rRYGB) has reported better WL results than LSG after LAGB (rLSG) in the literature. Nevertheless, rRYGB exhibited higher incidences of complications such as leaks and bleeding and increased rates of 30-day readmissions, reoperations, re-interventions, and extended operative durations compared to rLSG [9, 10]. However, good outcomes have also been reported after revisional OAGB (rOAGB), with a reported percentage of body mass index loss (%BMIL) of 33.17% at 5 years of follow-up and reported high rates of remission of associated medical conditions [11]. Moreover, high rates of GERD remission/improvement have been also reported reaching up to 81.7% after rOAGB [11]. All those revisional procedures were reportedly performed in one- or two-step approaches, with the safety of the one-step approach supported by data from systematic reviews and multi-center studies [12–14]. Currently, there is no consensus on what is the best revisional option after an unsuccessful LAGB. The available consensus statement reports RYGB, OAGB, and single anastomosis duodenal-ileal bypass with sleeve gastrectomy as accepted revision options. At the same time, the choice of the procedure should be individualized for every patient [15]. Therefore, further studies comparing the outcomes of different revisional options for unsuccessful LAGB are still needed to help surgeons choose the best option for every patient. In this study, we aimed to retrospectively assess the outcomes of three of the most performed one-step revisions after LAGB; rOAGB, rRYGB, and rLSG evaluate the technical feasibility, postoperative safety, and effects on WL, resolution of associated medical problems, and food tolerance over a 2-year follow-up (FU) period.

## Material and Methods

This retrospective database cohort study of rOAGB, rRYGB, and rLSG was performed as a one-step procedure for unsuccessful or complicated LAGB conducted at three hospitals (Department of Surgery at Medical Research Institute and Faculty of Medicine from Alexandria University, and Madina Women’s hospital, Alexandria, Egypt), between 2008 and 2019. The study was conducted in accordance with the principles of the Declaration of Helsinki and approved by the ethical committee board.

### Study Endpoints

WL was the primary endpoint assessed by the percentage of total weight loss (%TWL) and %EWL. The secondary endpoints included postoperative early and late complications and reoperations, improvement/resolution of associated medical problems, nutritional deficiencies, and food tolerance at 6 months, 1 year, and 2 years postoperatively.

### Inclusion Criteria

Patients with WR denote the recurrence of weight after an initial period of successful weight loss, defined as a 10% increase in weight compared to the nadir weight, or insufficient WL (IWL) refers to the inadequate loss of weight post-surgery, defined as unsuccessful to achieve a %EWL ≥ 50% after previous LAGB [16, 17]. Patients with band complications who had a one-step revisional surgery were included in this study.

### Exclusion Criteria

Patients who had a 2-step revision after LAGB, including all patients who planned for a one-step revision and converted to a two-step revision for visible band erosion during surgery, were excluded from this study.

### Pre-operative Workup

All patients underwent a preoperative upper GI endoscopy (UGE) to assess the stomach anatomy for band-related complications, including a tight or loose band, or diagnostics for gastroesophageal reflux disease (GERD) assessed by Los Angles (LA) classification [18], hiatal hernia (HH) and biopsy to exclude *Helicobacter pylori* (*H. pylori*) infection. Furthermore, all patients underwent routine abdominal ultrasound examinations to assess the need for concurrent cholecystectomy [19], and routine laboratory tests were performed in all cases.

### Data Collection

#### Preoperative Data

Baseline characteristics included age, sex, pre-operative lab tests, body mass index (BMI) (pre-band and pre-revision), nadir weight after band, the reason for revision, associated medical problems, pre-revision UGE findings, gallstones detected by ultrasonography, and food tolerance assessed by the one-page questionnaire [20].

#### Postoperative Data

Recorded data included operative time, hospital stay length, early and late postoperative complications, first 30-day readmissions and re-operations, WL parameters measured at 6 months, 1 year and 2 years FU, resolution/improvement of associated medical problems and post-operative lab tests at 2 years FU, post-operative UGE findings throughout FU, and post-operative food tolerance at 1 and 2 years FU [18]. Early complications were classified according to Clavien–Dindo (CD) classification [21].

### Multidisciplinary Team Assessment

A case-by-case multidisciplinary team (MDT) assessment was done to choose the revision procedure, discussing the reason for revision, the patient’s complaints regarding GERD, dysphagia, WL, UGE findings, the patient’s lifestyle and eating behavior, and associated medical problems. The revisional options were explained to the patients, showing the advantages and disadvantages of the procedures, including cost, long-term outcomes, and postoperative morbidities.

### Surgical Technique

The revisional procedures were performed by two independent high-volume surgeons (who operate on approximately 800 patients/year), using five standard ports. The complete surgical workflow for each procedure is presented in the Appendix. Concomitant operative procedures included crura repair for HH using unidirectional barbed 2/0 non-absorbable V-Loc sutures (Covidien, Mansfield, MA, USA) and cholecystectomy using the same ports without any additions. Mesenteric defect sides at the jejuno-jejunostomy and Petersen’s space were closed with non-absorbable V-Loc 2/0.

### Statistical Analysis

Descriptive and inferential statistics were used for the analyses. All data were tested for normality using the Kolmogorov–Smirnov, Q-Q plot, and Levene’s tests. Categorical variables are expressed as numbers and percentages. Normally and non-normally distributed continuous variables are presented as means with standard deviations (SDs) and medians with interquartile ranges. When appropriate, categorical variables were tested using Pearson’s chi-square or Fisher’s exact test. Normally distributed continuous data were tested with dependent samples using Student’s *t*-test for pre-and postoperative results. The Wilcoxon signed-rank test was used for skewed (nonparametric) data. Post hoc pairwise comparison between the study cohorts was performed through multiple Tukey pairwise comparisons, using a one-way analysis of variance (ANOVA) in three groups: group A (rRYGB vs. rOAGB), group B (rRYGB vs. rLSG), and group C (rOAGB vs. rLSG). Statistical significance was set at *p* < 0.05. Statistical analyses were performed using the R software, version 4.0.4 (R Foundation for Statistical Computing, Vienna, Austria).

### Sample Size Calculation

The sample size was calculated using the R software, version 4.1.3, and its “pwr” package based on a medium effect size of 0.25 for three comparison groups in one-way ANOVA and a power of 80% with an alpha of 0.05; this resulted in a minimum required sample size of 53 patients per group.

## Results

This study included 302 patients who underwent a revision for unsuccessful LAGB between 2008 and 2019 at two specialized bariatric centers. One patient per group was excluded due to band erosion. The rRYGB, rOAGB, and rLSG cohorts included 121, 97, and 81 patients.

### Lost to Follow-up Data

Forty-seven patients were lost to FU, including 19 (15.7%) rRYGB patients, 17 (18.0%) rOAGB patients, and 11 (13.6%) rLSG patients. The final analysis included 102, 80, and 70 patients in the rRYGB, rOAGB, and rLSG cohorts.

### Baseline Characteristics

The groups were similar regarding the demographic data, the weight loss pattern after the LAGB, and reasons for revision. There was a statistically significant difference between the study cohorts in weight before revision (*p*=0.034), the time between LAGB and revision (<0.001), and some lab tests such as hemoglobin (*p*=0.002), triglycerides (*p*<0.001), and vitamin B12 (*p*=0.033) levels. The incidence of associated medical problems before revision showed some statistically significant differences between the study cohorts, such as diabetes (*p*=0.024), dyslipidemia (*p*=0.004), sleep apnea (*p*<0.001), bronchial asthma (*p*=0.045), and cardiovascular diseases (*p*=0.017) (Table 1).

**Table 1:** Baseline characteristics

Characteristic	OAGB *N* = 80	RYGB *N* = 102	LSG *N* = 70	*P* value
Age (years), mean ± SD	42.6 ± 7.1	43.3 ± 7.0	43.6 ± 6.9	0.558
Sex (female), *n* (%)	69 (86.3%)	89 (87.3%)	61 (87.1%)	1.0
Mean ± SD				
Height (meter)	1.74 ± 0.1	1.70±.1.0	1.76 ± 0.1	0.096
Pre-band weight (kg)	136.2 ± 23.6	141.5 ± 27.9	141.4 ± 30.3	0.385
Pre-band BMI (kg/m^2^)	50.1 ± 9.0	50.7 ± 8.5	50.5 ± 9.7	0.903
Nadir weight after band (kg)	84.2 ± 17.3	86.1 ± 19.6	86.0 ± 19.4	0.782
Nadir BMI after band (kg/m^2^)	31.0 ± 6.4	30.9 ± 6.1	30.7 ± 6.0	0.960
Pre-revisional surgery weight (kg)	121.5 ± 19.0	130.3±23.8	123.3 ± 23.9	**0.034**
Pre-revisional surgery BMI (kg/m^2^)	44.8 ± 8.2	46.7±8.0	44.1 ± 7.7	0.451
Food tolerance pre-revision	20.6 ± 1.3	20.6 ± 1.4	20.5 ± 1.3	0.985
The time between the band and revisional surgery (years), mean ± SD	5.9 ± 1.3	8.6 ± 2.7	3.6 ± 0.5	**< 0.001**
Reason for revision *n* (%)				1.000
Dysphagia	10 (12.5%)	10 (12.8%)	11 (15.7%)	
Failure of the band	4 (5.0%)	4 (5.1%)	3 (4.3%)	
Infection of the band	1 (1.3%)	1 (1.3%)	0 (0.0%)	
Insufficient weight loss	10 (12.5%)	10 (12.8%)	9 (12.9%)	
Weight recurrence	55 (68.8%)	53 (67.9%)	47 (67.1%)	
Associated medical problem Pre-revision, *n* (%)				
Hypertension	11 (13.8%)	9 (8.8%)	14 (20.0%)	**0.067**
Diabetes	12 (15.0%)	5 (4.9%)	15 (21.4%)	**0.024**
Dyslipidemia	18 (22.5%)	37 (36.3%)	18 (25.7%)	**0.004**
Sleep apnea	0 (0.0%)	7 (6.9%)	13 (18.6%)	**< 0.001**
Bronchial asthma	0 (0.0%)	3 (2.9%)	5 (7.1%)	**0.045**
Cardiovascular disease	0 (0.0%)	8 (7.8%)	3 (4.3%)	**0.017**
Anticoagulant use	0 (0.0%)	2 (1.9%)	3 (4.3%)	0.168
Renal disease	0 (0.0%)	3 (2.9%)	1 (1.4%)	0.308
Steroid use	0 (0.0%)	3 (2.9%)	3 (4.3%)	0.140
Smoking	7 (8.8%)	7 (6.9%)	7 (10.0%)	1.0
Pre-revision lab investigations, mean ± SD				
Hemoglobin	13.6 ± 1.9	12.7 ± 1.7	12.7 ± 1.7	**0.002**
Ferritin	128.2 ± 71.0	120.0 ± 79.6	126.3 ± 55.3	0.744
WBC^§^	7.3 ± 2.2	7.4 ± 2.0	7.6 ± 1.8	0.792
SGOT^§^	20.9 ± 7.6	21.7 ± 8.1	22.0 ± 8.4	0.687
SGPT^§^	29.5 ± 12.0	27.7 ± 12.2	27.5 ± 11.9	0.524
UREA	27.1 ± 7.1	27.9 ± 7.7	28.4 ± 7.8	0.564
Creatinine	0.8 ± 0.2	0.8 ± 0.2	0.8 ± 0.2	0.901
INR	1.0 ± 0.1	1.0 ± 0.1	1.0 ± 0.1	0.973
T3	3.0 ± 0.8	2.9 ± 0.9	3.0 ± 0.9	0.778
T4	1.3 ± 0.5	1.2 ± 0.5	1.2 ± 0.6	0.553
TSH^§^	2.7 ± 1.3	2.6 ± 1.3	2.8 ± 1.3	0.807
Fasting blood sugar	96.3 ± 15.8	96.2 ± 17.5	97.7 ± 18.4	0.840
HbA1c^§^	5.0 ± 1.5	5.0 ± 1.4	5.1 ± 1.5	0.789
Total cholesterol	200.0 ± 67.4	198.7 ± 69.7	195.7 ± 70.7	0.927
Triglycerides	141.6 ± 30.1	175.1 ± 41.7	172.2 ± 39.8	**<0.001**
LDL cholesterol^§^	92.4 ± 41.3	97.2 ± 33.9	98.6 ± 34.3	0.544
Albumin gm/dl	4.0 ± 0.5	3.8 ± 0.7	3.9 ± 0.7	0.080
Calcium mg/dl	8.9 ± 1.1	8.7 ± 1.2	8.6 ± 1.2	0.319
Vitamin D ng/ml	34.1 ± 11.4	33.1 ± 12.6	34.2 ± 12.6	0.828
Vitamin B12 pg/ml	446.1 ± 240.0	392.3 ± 230.0	348.8 ± 208.0	**0.033**
PTH pg/ml^§^	37.6 ± 12.6	37.1 ± 12.6	37.8 ± 12.2	0.942
Preoperative endoscopy *n* (%)				
Slipped band/difficult passage of the endoscope	4 (5.0%)	10 (0.98%)	4 (5.7%)	0.168
Tight band	4 (5.0%)	4 (3.9%)	4 (5.7%)	1.0
Loose band	4 (5.0%)	4 (3.9%)	4 (5.7%)	1.0
Reflux				**<0.001**
GERD				
Grade A	33 (41.3%)	6 (5.9%)	1 (1.4%)	
Grade B	0 (0.0%)	4 (3.9%)	0 (0.0%)	
Grade C	0 (0.0%)	1 (0.98%)	0 (0.0%)	
Hiatal hernia	37 (46.3%)	5 (4.9%)	4 (5.7%)	**<0.001**
Helicobacter Pylori	8 (10.0%)	7 (6.8%)	7 (10.0%)	1
Dilated esophagus	16 (20.0%)	14 (13.7%)	12 (17.1%)	0.919

^§§^*WBC* white blood count, *SGOT* serum glutamic-oxaloacetic transaminase, *SGPT* serum glutamic-pyruvic transaminase, *TSH* thyroid-stimulating hormone, *HBA1c* hemoglobin A1C, *LDL* low-density lipoprotein, *PTH* parathormone

Bold: significance <0.05

The rOAGB cohort showed a statistically significant higher incidence of hiatal hernia (*p*<0.001) and grade “A” GERD (*p*<0.001) compared to the other cohorts. *H. pylori* was present in 10.0%, 6.8%, and 10.0% of patients in the rOAGB, rRYGB, and rLSG groups and was treated for 2 weeks with antibiotics, a proton pump inhibitor, and local gastro-protective agents. Complete eradication was confirmed by *H. pylori* antigen in stool tests.

BMI changes after LAGB were comparable in the study cohorts. Variations from the nadir to the pre-revision BMI were Δ+13.8, Δ+15.8, and Δ+13.4 kg/m^2^ in the rOAGB, rRYGB, and rLSG cohorts, respectively (*p*=0.674).

### Operative Data

There were significant differences in the operation time between the study cohorts (*p* < 0.001); the rOAGB had the shortest operation duration (85.6 ± 18.6 min), whereas the rRYGB had the longest (160.5 ± 34.6 min). The rate of concomitant HH repair was significantly higher in rOAGB than in rRYGB and rLSG (*p*<0.001). Of all the preoperatively diagnosed HHs, 75.6% were repaired during surgery (Table 2).

**Table 2:** Operative data outcomes

	OAGB *N* = 80	RYGB *N* = 102	LSG *N* = 70	*P* value
Operative time (min), mean ± SD	85.6 ± 18.6	160.78±34.20	124.5 ± 32.9	**<0.001**
Length of hospital stay (days), mean ± SD	2.0 ± 0.0	2.03±0.2	2.0 ± 0.2	0.233
Combined surgery *n* (%)				**<0.001**
CCC^§^	3 (3.8%)	7 (6.9%)	6 (8.6%)	
CCC and hiatal hernia repair	9 (11.3%)	0 (0.0%)	0 (0.0%)	
Hiatal hernia repair	28 (35.0%)	5 (4.9%)	4 (5.7%)	
BMI after revision, mean ± SD				
At 6 months	36.1 ± 7.5	34.5 ± 4.7	36.1 ± 5.4	0.185
At year 1	28.5 ± 4.2	27.2 ± 2.7	28.3 ± 3.0	**0.039**
At year 2	27.1 ± 3.0	26.8 ± 2.6	27.9 ± 2.3	0.061
Excess weight loss (%), mean ± SD				
At 6 months	46.1 ± 17.9	49.1 ± 13.7	42.3 ± 15.1	**0.034**
At year 1	84.7 ± 16.2	89.0 ± 12.9	81.4 ± 17.8	**0.014**
At year 2	91.2 ± 13.4	91.3 ± 14.1	83.6 ± 14.2	**< 0.001**
Total weight loss (%)				
Mean ± SD				
At 6 months	19.3±7.9	19.8±6.1	17.4±7.1	0.475
At year 1	35.5±7.6	37.9±8.6	34.4±10.9	0.568
At year 2	38.4±7.5	37.1±7.9	35.1±10.8	0.074
%TWL failure <20%				
At 6 months	53 (66.3%)	56 (54.9%)	63 (90%)	0.135
At year 1	1 (1.3%)	1 (0.98%)	8 (11.4%)	**0.001**
At year 2	0 (0.0%)	1 (0.98%)	6 (8.6%)	**0.001**
Associated medical problem				
Hypertension	***n*** **=11**	***n*** **= 9**	***n*** **= 14**	0.623
Resolution	7 (63.6%)	6 (69.2%)	8 (57.2%)	
Improvement	3 (27.3%)	3 (30.8%)	3 (21.4%)	
No change	1 (9.1%)	0 (0.0%)	3 (21.4%)	
Diabetes	***n*** **= 12**	***n*** **= 5**	***n*** **= 15**	1
Resolution	9 (75.0%)	3 (69.2%)	11 (73.3%)	
Improvement	2 (16.7%)	2 (23.1%)	3 (20.0%)	
No change	1 (8.3%)	0 (0.0%)	1 (6.7%)	
Dyslipidemia	***n*** **= 18**	***n*** **= 37**	***n*** **= 18**	0.616
Resolution	8 (44.4%)	22 (59.1%)	11 (61.1%)	
Improvement	8 (44.4%)	8 (22.7%)	4 (22.2%)	
No change	2 (11.2%)	7 (18.2%)	3 (16.7%)	
Food tolerance, mean ± SD				
After year 1	23.1 ± 0.9	23.0 ± 0.9	21.5 ± 1.3	**<0.001**
After year 2	23.1 ± 0.9	23.1 ± 0.8	22.2 ± 1.3	**<0.001**

§*CCC* concomitant cholecystectomy

Bold: significance <0.05

### Primary Outcome

#### BMI Changes

At 6 months and 1 and 2 years after revision, BMI was significantly reduced within the groups compared to the pre-revision BMI (rOAGB Δ−8.7, −16.3, −17.7 kg/m^2^; rRYGB: Δ−8.8, −16.1, −16.5 kg/m^2^; rLSG: Δ−8.0, −15.8, −16.2 kg/m^2^, respectively) (*p* ≤ 0.001). Significant differences were observed in BMI between the study cohorts at 1 year (the rRYGB group had the lowest BMI; *p* = 0.039) but not at 6 months or 2 years (*p* = 0.185 and 0.061, respectively).

#### %EWL and %TWL

**Fig. 1:**
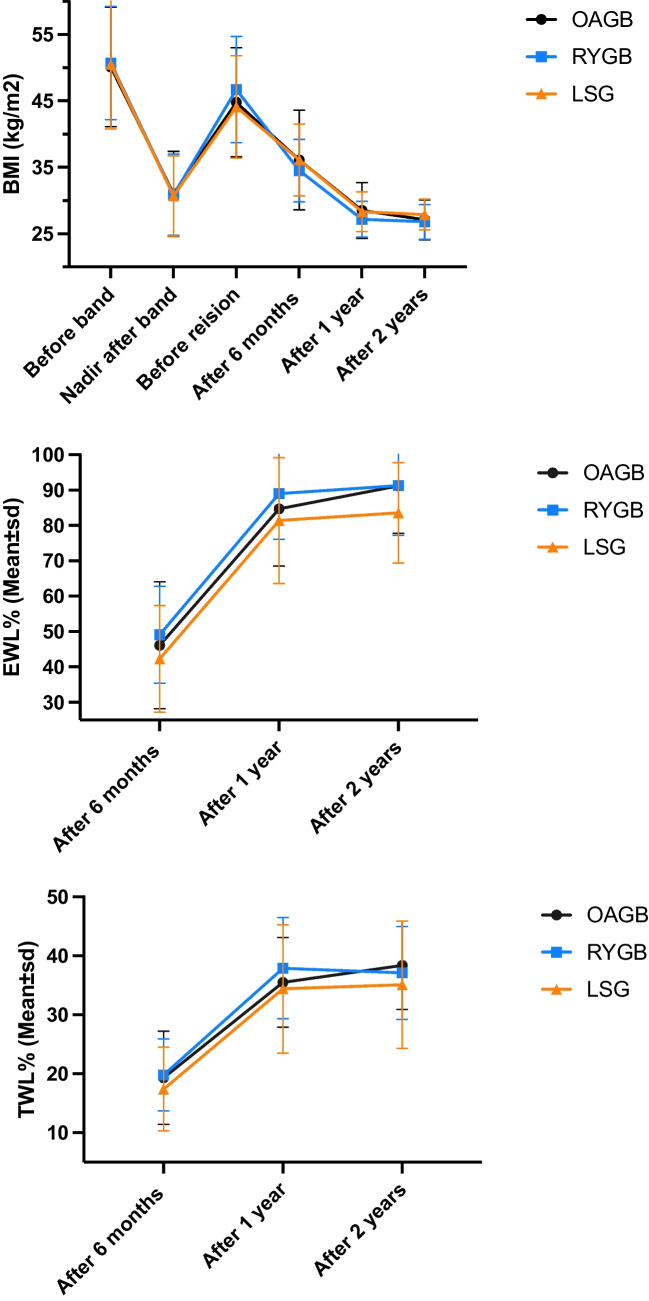
BMI changes, %EWL, and %TWL after revisional surgery

Significant differences in %EWL between the study cohorts were recorded at 6 months and 1 and 2 years after revision (*p* = 0.034, 0.014, and <0.001, respectively). rRYGB cohort had the highest %EWL, rOAGB, and rLSG. Conversely, %TWL was not significant at any of the three time points (*p* = 0.475, 0.568, and 0.074, respectively) (Fig. 1, Table 2).

#### Unsuccessful WR and WL

WR was recorded in four (5.7%) rLSG patients at 2 years FU. rRYGB and rOAGB cohorts showed no WR (*p*=0.001). Incidence of unsuccessful WL was significantly higher in rLSG at 1 and 2 years FU compared to rRYGB and rOAGB (*p*=0.001) (Table 2).

### Secondary Outcomes

#### Resolution/Improvement of Associated Medical Problems

There were no significant differences in the rates of resolution/improvement of associated medical problems between the study cohorts at 2 years FU (Table 2).

#### Postoperative Food Tolerance

**Fig. 2:**
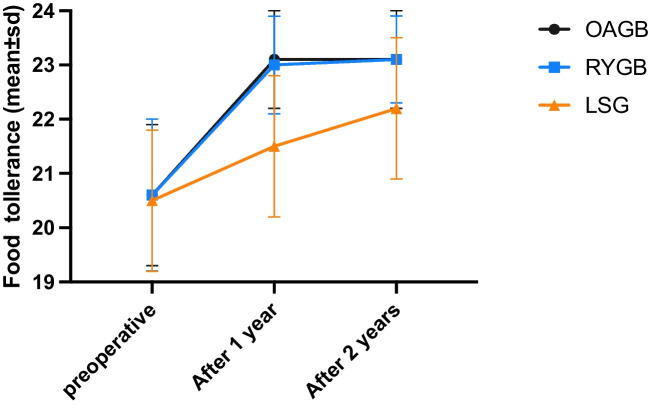
Food tolerance before and after revisional surgery

The food tolerance was significantly better in the rRYGB and rOAGB cohorts compared to that in rLSG at 1 and 2 years FU (*p* ≤ 0.001) (Fig. 2, Table 2).

#### Postoperative Early and Late Complications, Readmission, and Re-operation

Our findings indicated no postoperative leaks due to possible non-virginal tissues from adhesions and fiber-capsule around the anatomic side of the band. Regarding complications related to early re-operations, there was 1 (0.98%) intestinal obstruction and 2 (1.96%) bleedings with blood transfusion in rRYGB. Bleeding without blood transfusion was present in rOAGB (1.3%) and rLSG (2.8%) (*p*=0.198, 0.174). Overall, there were no significant differences among the study cohorts regarding early complications (*p* = 0.142) (Table 3).

**Table 3:** Post hoc pairwise surgical outcome comparisons between the three revisional surgeries (RYGB, OAGB, and LSG)

Outcomes	Group A RYGB-OAGB		Group B RYGB-LSG		Group C OAGB-LSG	
	Mean difference (95% CI)	*P*	Mean difference (95% CI)	*P*	Mean difference (95% CI)	*P*
Operative time (min)	74.9 (63.8, 85.9)	**< 0.001**	36.0 (24.6, 47.4)	**<0.001**	−38.9 (−50.2, −27.5)	**< 0.001**
Excess weight loss (%)						
At 6 months	2.9 (−3.0, 8.8)	0.472	6.8 (0.7, 12.8)	**0.026**	3.8 (−2.2, 9.9)	0.298
At year 1	4.3 (−1.6, 10.2)	0.204	7.6 (1.5, 13.7)	**0.011**	3.3 (−2.8, 9.4)	0.407
At year 2	0.1 (−5.1, 5.3)	0.998	7.7 (2.3, 13.1)	**0.003**	7.6 (2.2, 13.0)	**0.003**
Total weight loss (%)						
At 6 months	0.5 (−4.6,2.6)	0.247	2.4 (2.2,5.4)	0.744	1.9 (2.8,6.8)	0.942
At year 1	2.4 (2.8,6.8)	0.654	3.5 (2.4, 10.6)	0.241	1.1 −4.4, 8.8)	0.874
At year 2	−0.6 (−3.4, −1.8)	0.984	2.0 (−1.8,9.8)	0.364	3.3 (0.0,13.0)	0.435
Food tolerance						
After year 1	0.0 (−0.4, 0.4)	0.987	1.5 (1.1, 1.9)	**< 0.001**	1.5 (1.1, 1.9)	**< 0.001**
After year 2	0.0 (−0.4, 0.4)	0.996	0.8 (0.5, 1.2)	**< 0.001**	0.8 (0.4, 1.2)	**< 0.001**

Bold: significance <0.05

Late complications were higher in the rRYGB cohort but not significant with a marginal ulcer (MU)/melena (3.9% vs. 0%) (*p*=0.074). Late surgical complications were port side hernia in all three groups (1.3%, 0.98%, 1.4%) and one internal herniation in the RYGB (0.98%) (Table 4).

**Table 4:** Postoperative data

	OAGB *N* = 80	RYGB *N* = 102	LSG *N* = 70	*P* value
Early re-operation	1 (1.3%)	3 (2.9%)	2 (2.9%)	1.000
Intestinal obstruction	0 (0.0%)	1 (0.98%)	0 (0.0%)	
Bleeding with blood transfusion				0.198
*Bleeding from omentum*	0 (0.0%)	1 (0.98%)	0 (0.0%)	
*Bleeding from the port site*	0 (0.0%)	1 (0.98%)	0 (0.0%)	
Bleeding without blood transfusion				
*Bleeding from omentum*	1 (1.3%)	0 (0.0%)	1 (1.4%)	0.174
*Bleeding from the port site*	0 (0.0%)	0 (0.0%)	1 (1.4%)	
Early complications				0.142
MVO^§^	1 (1.3%)	3 (2.9%)	0 (0.0%)	
Melena	0 (0.0%)	3 (2.9%)	0 (0.0%)	
UTI^§^	0 (0.0%)	0 (0.0%)	2 (2.9%)	
Wound infection	1 (1.3%)	2 (1.9%)	0 (0.0%)	
Clavien-Dindo classifications	**3 (3.75%)**	**11 (10.8%)**	**4 (5.7%)**	0.754
CD I	0 (0%)	3 (2.9%)	0 (0%)	
CD II	1 (1.3%)	2 (1.9%)	2 (2.9%)	
CD IIIb	1 (1.3%)	3 (2.9%)	2 (2.9%)	
CD IVa	1 (1.3%)	3 (2.9%)	0 (0.0%)	
Late complications				0.074
Marginal ulcer/melena	0 (0.0%)	4 (3.9%)	0 (0.0%)	
Late re-operation				
Port site hernia	1 (1.3%)	1 (0.98%)	1 (1.4%)	
Internal Herniation	0 (0.0%)	1 (0.98%)	-	
Endoscopy after 2 years (selective), *n* (%)	**8 (10%)**	**8 (10.3%)**	**39 (55.7%)**	**0.002**
Hiatal hernia de novo	1 (1.3%)	0 (0%)	19 (27.1%)	**0.014**
Marginal ulcer	3 (3.8%)	2 (2.6%)	–	
Reflux^±/±±^	4 (5.0%)^±^	0 (0%)	20 (28.6%)^±±^	
Stenosis	0 (0%)	4 (5.1%)	0 (0%)	
Stenosis and reflux	0 (0%)	2 (2.6%)	0 (0%)	

*MVO* mesenteric vascular occlusion, *UTI* urinary tract infection

^±^Bile reflux

^±±^Acid Reflux

Bold: significance <0.05

#### Postoperative Clavien-Dindo Classification

There were no significant differences in the CD classification among the study cohorts.

#### Postoperative Endoscopy

The rate of selective endoscopy upon patient complaints at 2 years FU was significantly higher in rLSG compared to that in rRYGB and rOAGB (*p* = 0.002) (Table 4). Moreover, there was a significant difference in the rates of endoscopic findings between the study cohorts, mainly in the rates of de novo HH and reflux (*p* = 0.014) (Table 4).

#### Postoperative Nutritional Deficiencies

Significantly higher rates of anemia and deficiencies in calcium, vitamin D, vitamin B12, and albumin were recorded in the rOAGB at 2 years FU compared to pre-operatively (Table 5).

**Table 5:** Nutritional deficiencies before and after revisional surgery

	OAGB *N***=**80			RYGB *N*=102			LSG *N*=70		
Deficiency	Before	After	*P*	Before	After	*P*	Before	After	*P*
Ferritin < 30, *n* (%)	0 (0.0%)	4 (5.0%)	0.134	4 (3.94%)	8 (7.8%)	0.387	0 (0.0%)	7 (10.0%)	**0.023**
Calcium < 8.6, *n* (%)	31 (38.8%)	48 (60.0%)	**0.001**	34 (33.3%)	26 (25.5%)	0.256	32 (45.7%)	23 (32.9%)	0.151
Vitamin D < 20, *n* (%)	10 (12.5%)	19 (23.8%)	**0.027**	14 (17.9%)	14 (17.9%)	1.000	10 (14.3%)	10 (14.3%)	1.000
Vitamin B12 < 200, *n* (%)	7 (8.8%)	23 (28.8%)	**<0.001**	14 (13.7%)	10 (9.8%)	0.540	19 (27.1%)	12 (17.1%)	0.190
Albumin < 3, *n* (%)	0 (0.0%)	20 (25.0%)	**<0.001**	12 (11.8%)	8 (7.8%)	0.423	9 (12.9%)	7 (10.0%)	0.773
Hemoglobin < 11, *n* (%)	7 (8.8%)	27 (33.8%)	**< 0.001**	12 (11.8%)	20 (19.6%)	0.170	10 (14.3%)	19 (27.1%)	0.095
Outcomes^±±^ After revisional surgery	Group A RYGB-OAGB			Group B RYGB-LSG			Group C OAGB-LSG		
	Delta deficiency *n***=**	*P*		Delta deficiency *n***=**		*P*	Delta deficiency *n***=**	*P*	
Ferritin	+4	0.245		−1		0.884	−3	0.274	
Calcium	−22	**<0.001**		−3		0.247	+25	**<0.001**	
Vitamin D	−5	0.067		−4		0.297	+9	**<0.001**	
Vitamin B12	−13	**<0.001**		+2		0.467	+11	**<0.001**	
Albumin	−12	**<0.001**		−1		0.984	+13	**<0.001**	
Hemoglobin	−7	**0.004**		−1		0.974	+8	**0.002**	

^±±^Post hoc pairwise surgical outcome comparisons between the three revisional surgeries (RYGB, OAGB, and LSG)

Bold: significance <0.05

There were no significant changes in the nutritional deficiency statuses after 2 years in the rRYGB and the rLSG cohorts, except for a higher rate of ferritin deficiency in rLSG (*p* = 0.023) (Table 5).

### Post Hoc Pairwise Comparisons Between the Three Revision Groups (Table 3)

#### Group Comparisons of Operation Time

There were significant differences in the operation time between all groups (*p* < 0.001).

#### Group Comparisons of BMI and %EWL

The rRYGB cohort had a lower mean BMI by 1.3 kg/m^2^ (95% confidence interval [CI]: −2.6–0.0, *p* = 0.042) at 1 year.

In group A (rRYGB vs. rOAGB), the %EWL did not significantly differ at 6 months (2.9%), 1 year (4.3%), and 2 years (0.1%) (*p* = 0.472, 0.204, and 0.998 respectively).

In group B (rRYGB vs. rLSG), the RYGB group had significantly higher %EWL at all three FUs than the LSG group (6.8%, 7.6%, and 7.7%; *p* = 0.026, 0.011, and 0.003 respectively).

In group C (rOAGB vs. rLSG), the OAGB group had significantly higher %EWL than the LSG group at 2 years (7.6%) (*p* = 0.003). No significant differences existed between the %TWL in groups A, B, and C (Table 3).

#### Group Comparisons in Food Tolerance

Group A showed no significant differences in food tolerance at 1 and 2 years (*p* = 0.987 and 0.996). Conversely, groups B and C had significantly greater food tolerance differences compared with group A (groups B and C, 1.5, 0.8) (*p* < 0.001) (Table 3).

#### Group Comparisons of Nutritional Deficiencies

The rOAGB had significantly higher rates of nutritional deficiencies in groups A and C. No significant differences were observed between the rRYGB and rLSG cohorts (Table 5).

## Discussion

In this study, we evaluated the outcomes of rOAGB, rRYGB, and rLSG through 2 years of FU as revisional options for unsuccessful LAGB. rOAGB and rRYGB had better %EWL than rLSG, while rOAGB and rLSG had lower CD ≥ 3 complication rates. Only rLSG showed WR at 2 years FU and had significantly higher rates of unsuccessful WL at 1 and 2 years FU than rOAGB and rRYGB.

### Revisional Surgery after LAGB

Different indications have been reported in the literature for revision after LAGB, primarily non-responders having WR or insufficient WL and band-related complications such as GERD, dysphagia, band erosion, band slippage, dilated pouch, and port infection [8, 14, 22]. A nationwide cohort study found that 70.4% and 85.5% of rRYGB and rOAGB were non-responders, while 29.6% and 14.5% had band-related problems, respectively [22]. This study showed corresponding results regarding the indications of revision, with WR and insufficient WL forming around 80% of the indications for revision. Band removal alone for complications is associated with WR; Aarts et al. reported complete WR 5 years after band removal without additional surgery [7]. This raises the significance of having a revisional procedure also for band-related complications instead of band removal alone.

While LAGB is no longer a popular procedure, a recent study by Nasri et al. has reported LAGB to remain a safe and durable bariatric option. However, they reported a mean %EWL of 42.25% and a re-operation rate of 20.25% for insufficient WL over a mean FU period of 5.78 years [23].

Past research has examined various aspects of revisional surgery after band placement. For instance, a systematic review from 2017 focused on revisional RYGB and LSG post-band removal [8]. Another study investigated OAGB as a tertiary procedure and provided long-term follow-up data, albeit with limited statistical power [24]. A recent study examined three revisional cohorts (OAGB, RYGB, LSG) but also suffered from low statistical power in the OAGB arm [6]. Notably, none of these studies performed inter-group comparisons regarding nutritional status and 2-year outcomes.

#### %EWL and %TWL

In this study, rRYGB and rOAGB had equal %EWL at 2 years FU reaching >90% (*p* = 0.998), significantly higher than rLSG with a %EWL of 83.6% (*p* < 0.001). OAGB has well-reported high safety and efficacy as a primary or revisional bariatric procedure [11, 25–27]. OAGB has been recognized as an effective bariatric procedure with an average %EWL of 78% reported at 2 years FU in a systematic review of 12,807 OAGB and rOAGB patients [26]. rRYGB and rLSG have been extensively reported after unsuccessful LAGB with better weight loss in rRYGB than rLSG at 2 years FU [8–10, 28, 29]. Conversely, one study reported no significant WL difference between rRYGB and rLSG throughout 5 years of FU [30]. rOAGB has demonstrated higher long-term %EWL and lower rates of insufficient WL than rRYGB throughout the longer FU (>5 years) [22]. Moreover, in a recent study, rOAGB showed significantly higher %EWL than rLSG at 2 (70.4% vs. 55.6%) and 4 years (68.7% vs. 54.6%) [31]. A 2023 study [32] delineated a noticeable difference between primary RYGB and revisional RYGB post-LAGB, with significantly lower %EWL (93.7 vs. 64.1%) and %TWL (47.3% vs. 24.6%) after 2 years. However, it is to be noted that this outcome was exclusively observed in revisional RYGB procedures. A 2022 study [33] investigated long-term outcomes over 8 years, revealing that revisional surgery groups exhibited significantly poorer performance in weight loss (EWL 67 vs. 53, TWL 34 vs 26). However, the sample size for this long-term follow-up was notably small, with results obtained from 8 and 10 patients, respectively. A 2021 study [34] compared primary RYGB and revisional RYGB post-LAGB, with the former demonstrating superior EWL outcomes (73.0 vs 62.4%). Interestingly, the results favored revisional surgery when comparing primary LSG and revisional LSG post-LAGB (54.8 vs. 60.2%). In our study, WR at 2 years FU was recorded only in rLSG in 4 (5.7%) patients. Three also had WR, and the fourth had insufficient WL after the primary LAGB. At 6-month FU, >50% of patients of the study cohorts were unsuccessful in achieving %TWL ≥ 20%. At 1 and 2 years, rLSG had significantly higher rates of unsuccessful WL (< 20 %TWL) (11.4% and 8.6% respectively) than rOAGB and rRYGB, who experienced rates of unsuccessful WL of 0% and 0.98% respectively at 2 years FU. After BMS, revision procedures may be the best option for insufficient WL or WR. Even though revisional surgery can produce lower WL than primary, WL outcomes are still reported as successful after multiple procedures. A study from Raglione et al. reported a %TWL of 29.6% and a %EWL of 53.4% after 60 months FU following a third or more BMS.

#### Complications

In our study, no leaks were observed. rRYGB had higher complication rates according to CD classification (10.8% vs. 3.75% and 5.7% in rOAGB and rLSG, respectively, *p*=0.754), and re-operations were not statistically significant. The smaller pouch of the rRYGB might require dissection and stapling in the region of the fibrous capsule formed around the band, which might increase the incidence of complications such as leaks and bleeding. In contrast, in the rOAGB, the gastric pouch is much longer, and creating the gastrojejunostomy in the fibrous tissue could be easily avoided. Similarly, in rLSG, stapling through the fibrous tissue could be easily avoided.

Lower rates of intra-abdominal complications have been reported in rOAGB than rRYGB after restrictive procedures (1.1% vs. 4.9% respectively, *p* = 0.025), along with significantly higher %TWL and %EWL at 1 and 2 years FU [30]. Some other studies reported no significant differences in complication rates between rOAGB and rRYGB [22, 35]. Some meta-analyses and nationwide data analysis studies have reported higher rates of complication rRYGB than rLSG [9, 10]. Conversely, data from two recent meta-analyses reported similar rates of complication rLSG and rRYGB [8, 29]. Considering the WL outcomes, rLSG reportedly had less WL outcomes; however, it is still a popular option for its high safety profile compared to the rRYGB, which allegedly has better WL outcomes, while with the rOAGB gaining popularity, it might be the best option given the high WL outcomes and higher safety compared to rRYGB.

This study identified one internal herniation case in the RYGB group, consisting of 102 patients with complete follow-up data. In comparison, no instances of internal herniation were found in the OAGB group. To put these findings into context, a 2020 study [36] reported an incidence rate of internal herniation at 1.3%. Applying this rate to our RYGB cohort of 102 patients suggests the potential for 1 to 2 cases of IH, aligning with our observed data.

Nonetheless, it is crucial to recognize the constraints inherent to our study. The 2-year follow-up duration qualifies as a “mid-term” assessment, between “short-term” durations of less than 2 years and “long-term” durations extending beyond 5 years. Our retrospective study benefitted from the most comprehensive and accurate data available within these 2 years, demonstrating fewer participant dropouts and more complete data sets than would likely have been available in a study with more extended follow-up duration. Given that the mean time from surgery to IH in the referenced 2020 study was 17.98 ± 11.2 months, it is plausible that additional cases of IH may not have manifested within our 24-month follow-up window.

#### Post-operative UGE Findings

Post-operative UGE was performed for patients with symptoms suggestive of upper GI pathology, such as dyspepsia, reflux, vomiting, or melena. The rate of UGE was significantly higher in rLSG cohort (*p*=0.002) with high rates of de novo hiatal hernia (27.1%) and acid reflux (28.6%). Bile reflux was seen in 5% of the rOAGB cohort, and all were managed conservatively. Similar reflux rates in rOAGB and rLSG have been reported recently [28]. Bile reflux is a feared problem after OAGB. However, lower incidences of bile reflux (0.4–1.8%) were reported in large OAGB series [25, 37, 38]. Conversion of OAGB to RYGB for bile reflux has been studied in a series of 2780 OAGB patients; of them, 1.2% needed conversion to RYGB with a 93.8% GERD resolution rate [38]. Conversion to RYGB is a well-reported option for persistent bile reflux after OAGB [39].

A higher incidence of pre-operatively diagnosed HH was seen in rOAGB (46.3%) in this study, compared to 4.9% and 5.7% in rRYGB, and rLSG, respectively. The presence of HH, regardless of size, should not be considered a contraindication for OAGB, as stated by the IFSO Consensus Conference Statement on OAGB in 2020 [40]. However, post-operative de novo HH incidence was only 1.3% in rOAGB, while it was significantly higher in rLSG as diagnosed by UGE. UGE was only done for patients having complaints; this may explain the higher incidence of HH in rLSG, as the patient complaint ratio was significantly lower in the rOAGB and rRYGB cohorts. Moreover, the anatomical differences in the procedures with excessive dissection at the diaphragmatic crura and hiatus may explain this higher HH rate in rLSG. LSG has reported high rates of HH, reaching up to 84.6% at > 18 months FU [41]. Thus, LSG is better avoided in patients with pre-operatively diagnosed HH who might get better outcomes with OAGB or RYGB.

Marginal ulcers (MU) occurred in 3.8% (rOAGB) and 2.6% (rRYGB). Higher rates have been reported after rOAGB (17.6%) and rRYGB (9.5%) [42]. The larger pouch of OAGB leads to more acid secretion and acid exposure than RYGB, which, besides the bile exposure, may explain a higher incidence of MU in OAGB. Medical treatment was effective in both groups, and none of our patients required another revision during FU. Moreover, selective UGE for symptomatic patients only might have led to underestimated rates of MU in this study.

#### One- vs. Two-Step Procedures

Only one-step procedures were included in this study. Intraoperative checks were performed for safety. Three patients were excluded for intra-operative identification of band erosion and underwent a two-step procedure. One-step procedures minimize operations and hospital admissions and reduce costs. Higher risk of complications might be expected in one-step revision due to fibrotic tissue at the band site, especially with concomitant band complications. However, several single- and multi-center studies and meta-analyses have reported the high safety profile of one-step rOAGB, rRYGB, and rLSG for unsuccessful LAGB that is comparable to the two-step procedures and even primary procedures [12–14, 26, 43, 44].

A recent meta-analysis in 2020 reported an equal overall leakage rate between one- and two-step revisions after LAGB and suggested improved safety of one-step procedures in rRYGB and of two-step procedures in rLSG [45]. However, a comment published by Gagner noted some possible funnel-plot bias in that meta-analysis [46]. Moreover, a recent study by Spaniolas et al. that included 4330 patients reported lower morbidity in one-step than the two-step approach, in addition to fewer complications and lower readmission, in favor of rLSG over rRYGB [47]. Upon evaluating the methodology and statistical approach and analyzing the power and results of both studies, Spaniolas et al. provided a better foundation and results for a one-step revisional surgery. Our study also confirmed this with low CD scores with a CD ≥ 3 recorded in 2.6%, 5.8%, and 2.9% of rOAGB, rRYGB, and rLSG, respectively (*p* = 0.754), low readmission and re-operation rates, and no mortality.

The decision to simultaneously remove the band and perform a one-step vs. two-step revision surgery with variable intervals is complex. Nevertheless, our results and other studies indicate that the one-step strategy is safe. After intraoperative evaluation, the surgeon must re-evaluate the situation to determine the best approach.

#### Associated Medical Problems

Comparable rates of associated medical conditions’ resolution were recorded in the three cohorts of this study. A recent study has reported a 7 times higher improvement of associated medical problems after rRYGB than rLSG [34].

However, a systematic review reported similar rates of associated medical problem resolution among rRYGB and rLSG, with pooled resolution rates of 46.5% and 35.9% for diabetes and hypertension, respectively [8]. Another review on rOAGB reported higher rates of resolution of diabetes and hypertension, reaching 80.5%, and 63.7%, respectively [26].

#### Food Tolerance

While all our study cohorts had significant improvement in food tolerance compared to pre-revision, rOAGB and rRYGB had significantly better food tolerance than rLSG (*p* ≤ 0.001), with no significant differences between rOAGB and rRYGB (*p* = 0.987). Although the food tolerance worsened after LSG, WL was still superior to the preoperative situation.

When food tolerance improves, patients can gain more weight over time. The release of restrictions on the pouch and low-pressure system of rRYGB and rOAGB can improve food consumption. Similarly, gastric dilatation after rLSG would increase food tolerance scores and possibly lower WL or lead to WR over time. Monitoring food tolerance scores can help signal WR in those post-revision patients.

#### Nutritional Effects

rOAGB had significantly higher rates of nutritional deficiencies compared to the other two cohorts of this study in calcium, vitamins D and B12, and albumin and hemoglobin levels; however, none of the patients required readmission due to malnutrition. Nutritional deficiencies after OAGB are well reported in the literature, with reported significant hypoalbuminemia and anemia compared to LSG at 1 year FU and reported hypoproteinemia, hypoalbuminemia, anemia, and hypocalcemia compared to RYGB [48, 49]. The systematic review and meta-analysis on nutritional complications following OAGB yielded findings consistent with our study [49]. In our clinic, the gastro-jejunostomy is constructed 200 cm from the ligament of Treitz for revisional cases to optimize patient outcomes since it is known that revisional surgery is known with inferior weight loss compared to primary BMS [8, 24, 33, 50]. For primary cases, we have recently transitioned to a 150-cm approach. This change was influenced by findings from a study by Bertrand et al., which suggested comparable weight loss results and a potentially reduced risk of malnutrition with this length [51]. Therefore, emphasizing long-term nutritional follow-up, ensuring patient compliance with dietary supplements after rOAGB, and considering this complication when performing rOAGB is crucial.

#### Surgical Technique

The appropriate BL and AL lengths to be used remain controversial. Data from meta-analyses showed increased WL using a longer BL; however, other studies showed no differences between shorter and longer BLs [52–54]. A longer BL is reported to increase the need for supplementation with vitamins B12, A, and folic acid [54]. A long BL (200 cm) with a short AL (60 cm) in RYGB increased the WL effect compared to a short BL (60 cm) with a long AL (150 cm); however, this resulted in significantly greater malabsorption and need for supplementation [55]. For OAGB, some authors used a fixed BL length of 200 cm [56], whereas others recommended a BL length of 150 cm to avoid severe nutritional deficiencies [57]. In addition, tailoring the BL in RYGB and OAGB considering patient BMI has been described [58]. This study showed that BL and AL lengths achieved significant %EWL and positive nutritional values.

### Limitations

This study has some limitations. The 2-year follow-up is relatively short, and longer follow-up may reveal other changes regarding the WL, long-term complications, and resolution of associated medical conditions. Furthermore, more variables, such as gut hormone levels, body composition, and preoperative data before the primary procedure, could help improve the predictions.

## Conclusion

One-step revision is safe after LAGB. rOAGB and rRYGB have the best outcomes after unsuccessful or complicated LAGB compared to rLSG in terms of WL, food tolerance, technical feasibility, and safety. Strict dietary supplements are advised after revisional surgery, especially the rOAGB had significantly higher rates of nutritional deficiencies.

## Data Availability

Data is available with the corresponding author.

## Appendix

### Surgical Workflow Per Procedure

All surgeries in our cohort were performed in a single-step conversion standard; five ports were used, including three 12-mm ports (for the camera and right and left working ports) and two 5-mm ports (for liver retraction and the assistant). Pneumoperitoneum was created after using optical trocars for entry. Adhesions around the stomach were dissected using the energy device EnSeal® (Ethicon Endo-Surgery, Cincinnati, OH, USA). Subsequently, the tube was disconnected at the level of the abdominal wall. Adhesions between the ventral aspect of the stomach and liver were removed to ensure optimal placement of the liver retractor. The entire scar capsule of the band was dismantled, keeping in mind that the band could have been placed “pars flaccida” or “perigastrically.” The entire band was then placed aside.

### LAGB to RYGB

The gastric pouch was created from 5 to 6 cm below the esophagogastric junction using an Echelon Flex Endopath 60-mm linear stapler (Ethicon Endo-Surgery, Cincinnati, OH, USA) over a 40-Fr bougie using gold and blue reloads. Whenever possible, we tried to create the pouch above the level of the band. The fibrous band capsule was routinely removed before stapling; however, some scarring in the gastric tissue may exist in the previous LAGB site after capsule removal. Residual scarred gastric tissue was encountered in less than 20% of patients, and placing the stapler above the scarred tissue was possible in most of them. Stapling on scarred tissue was done using green and black reloads to create the pouch. The same stapler was used for the construction of the gastrojejunostomy and jejunojejunostomy using blue and white reloads, respectively, with equal 100-cm biliopancreatic and alimentary limbs, starting in 2016 (cases operated before 2016 had a 60-cm biliopancreatic limb and 150-cm alimentary limb). The stapling defects were closed in two layers using barbed 3/0 V-Loc 180 sutures (Covidien, Mansfield, MA, USA). The staple line in the gastric pouch and remnant stomach was reinforced with continuous seromuscular sutures using the same barbed sutures. All mesenteric defects were closed using 3/0 V-Loc nonabsorbable sutures (Covidien, Mansfield, MA, USA).

### LAGB to OAGB

The long gastric pouch was started at the crow’s foot on the lesser gastric curvature over a 40-Fr bougie using gold and blue reloads. Blue reloads were used for gastro-jejunostomy construction, 200 cm from the ligament of Treitz. The long gastric pouch allowed performing anastomosis in healthy gastric tissue distal to the band scar. Stapling defect closure and staple line reinforcement were performed as in RYGB. Closure of the mesenteric defects was not required in the OAGB group.

### LAGB to LSG

Approximately 70–80% of the gastric volume was resected over a 40-Fr bougie using gold and blue reloads. We avoided stapling over the scarred gastric tissues at the site of the removed band capsule, and in some cases we made the stapling at the angle of His two cm lateral to the esophagogastric junction keeping the esophago-gastric junction region wide to be narrowed when needed by invaginating barbed sutures during staple line reinforcement.

Regardless of the type of conversional procedure, crural repair for diagnosed hiatal hernia using 2/0 V-Loc non-absorbable sutures (Covidien, Mansfield, MA, USA) was attempted in the three study groups. Concomitant cholecystectomy was performed in all patients with pre-operatively diagnosed calcular cholecystitis. Additionally, an intraoperative methylene blue leak test was routinely performed, and a tube drain was placed in the left subphrenic space. Finally, the gastric band was exteriorized and ports removed.
